# The female journey following sexual assault: Medical care, law enforcement and judicial outcomes

**DOI:** 10.1002/ijgo.71190

**Published:** 2026-06-30

**Authors:** Vanita Parekh, Michael Woodburn, Stephanie Leonard, Bridget Bourke, Janine McMinn, Anna Brkic, Ash Byron, Katelyn Pomroy

**Affiliations:** ^1^ Clinical Forensic Medical Services Canberra Health Services Canberra Australian Capital Territory Australia; ^2^ The Australian National University Canberra Australia; ^3^ University of Canberra Canberra Australia; ^4^ ACT Policing, Australian Federal Police Australia

**Keywords:** legal outcomes following sexual assault, rape, sex offences conviction, sexual assault

## Abstract

**Objective:**

Sexual violence affects 22% of Australian women, posing health and justice challenges. Systemic failures, fragmented data, and low conviction rates hinder effective responses. This study integrates Australian Capital Territory (ACT) medical and policing datasets to examine victim characteristics and case progression from clinical presentation to legal outcomes.

**Methods:**

This retrospective study analyzed 2179 deidentified cases of female sexual assault victims presenting to Forensic and Medical Sexual Assault Care (FAMSAC) between 2004 and 2022. De‐identified patient variables and ACT Policing data were integrated and analyzed to examine victim characteristics and case progression from medical consultation to legal outcomes.

**Results:**

Of 2179 females presenting for forensic and medical care, 41.8% reported to ACT Policing, 19.2% progressed to court and 10.4% of cases resulted in a conviction. Drug use (14.9%) was negatively associated with case progression, while alcohol use and pre‐existing mental health conditions showed no significant effect. No victim, assailant, or medical factors were associated with conviction outcomes. Among cases withdrawn at the policing stage, anal penetration, forensic photography, and presentation outside business hours were associated with continuation to court. Overall, 54.3% of cases that proceeded to court resulted in conviction.

**Conclusion:**

Through collaborative practices, this study establishes a baseline for continuous system review from forensic medical presentation to court outcome. Legal outcomes were independent of victim characteristics, assault details, and medical findings, with attrition at every stage. Medical documentation alone could not predict conviction, though it may support the finders of fact. Further research is needed to assess whether medical evidence influences police action, court pleas, and convictions.

## 1 INTRODUCTION

In Australia, 22% of women have experienced sexual violence since the age of 15^1^; the adverse reproductive,^2^ sexual,^3^ physical,^4^, ^5^ and psychosocial effects^6^, ^7^ of sexual violence are a public health emergency.^8^ Beyond these consequences, sexual violence generates complex challenges within the medical, law enforcement and legal systems that directly impact victims, including low trust, re‐traumatization, and low conviction rates.^9^ Systemic failures in response to sexual violence have been described for medical, law enforcement and prosecution systems in the Australian Capital Territory (ACT). The same report describes the need for a whole‐of‐system review and the absence of a unified dataset, which has hindered case tracking of the ACT system response to sexual violence.^10^ Case tracking in the ACT should be a straightforward exercise, as the ACT is a contained geographical metropolitan jurisdiction within New South Wales (NSW). Without measurement in an established dataset, the capacity for improvement will continue to be elusive.^11^

Other jurisdictions have been able to measure and analyze legal outcomes; however, medical evidence at its best can only corroborate a victim's testimony.^12^ No two cases are the same, but there are validated ways of responding to sexual assault using the victim‐centric approach to medical care^13^ and investigation,^14^ with explanations for the lack of victim injury,^15^ and delayed reporting.^16^, ^17^

Research on legal outcomes and the impact of medicolegal evidence in sexual violence cases remains limited, with few international comparative studies^18^ and scarce Australian data.^19^ The ACT's Forensic and Medical Sexual Assault Care (FAMSAC) has provided care after sexual assault since 2001, with the model of medical care,^20^ sexually transmitted infections,^21^ and medical and legal outcomes for males previously described.^22^ FAMSAC is the only provider of forensic medical evidence collection in the ACT jurisdiction. ACT Policing provides law enforcement services in the jurisdiction and briefs of evidence to the ACT Office of the Director of Public Prosecutions (ODPP). The aim of the present study was to derive insights from the deidentified pooled data from FAMSAC and ACT Policing, exploring victim characteristics and case progression from medical consultation to conviction outcomes.

## 2 MATERIALS AND METHODS

### 2.1 Study population and ethical approval

This retrospective clinical audit comprised all 2179 female patients who presented to FAMSAC between January 2004 and October 2022. Changes to clinical forensic evidence collection protocols and process were minimal over this time period. These data and patient consent are reported under ethical approval ACT reference 2023.LRE.00009. REGIS reference 2022/ETH02749.

### 2.2 Model of care at FAMSAC

FAMSAC provides medical care with the option of forensic assessment after sexual assault to individuals, regardless of gender. Although this study only describes female patients who have undergone menarche, male patients were also audited over this time period and are described elsewhere.^22^ FAMSAC doctors and nurses provide patient care at a designated medical facility to those in the ACT and surrounding geographical region on a 24‐h, 7‐day‐a‐week basis. During the study period the principal forensic medical examiner was a medical practitioner. Immediate medical care and follow‐up is offered to all patients, regardless of their decision about forensic assessment or an intention of reporting to ACT Policing. Consent for injury documentation, photography, DNA and toxicology is obtained from the individual prior to the examination and is detailed in the medical examination record. Genital photography is not undertaken by FAMSAC due to concerns regarding data storage and distribution and the significance of genital injuries in adults. Forensic medical examination is offered where there is the potential for evidentiary findings (e.g., injury, DNA, or toxicology) within the relevant forensic timeframes.

When forensic assessment is undertaken, forensic photography and medical testing for sexually transmitted infections occurs after the collection of forensic specimens for DNA analysis. Sequencing of the forensic medical examination and other measures are undertaken to minimize the chance of DNA cross contamination.

Additional medical management is tailored to the patient's requirements, such as treatment of genital and non‐genital injuries. Medical care, treatment, and forensic collection are provided at no cost to the patient.

### 2.3 Clinical and investigation outcome data collection

At the time of each case, the attending doctor or nurse completed a summary datasheet for each patient who accesses FAMSAC services following sexual assault. Data were entered into a password protected MS Excel spreadsheet stored on secured hospital servers, with access restricted to study researchers and with retention for 10 years in accordance with ethics approval; cases were then matched to the ACT Policing dataset. Both datasets were de‐identified by using statistical linkage key (SLK‐581) coding before matching.^23^ Once the criminal justice outcome was validated in the ACT Policing data, that result was compared with FAMSAC‐collected victimology and offense particulars. Ethical and ACT Policing approvals for the research were obtained prior to any de‐identified data matching. For cases with missing clinical data, clinical files were re‐examined; however, the missing data could not be resolved.

During this research, the authors conducted a manual case tracking review of all sexual assault matters where complainants reported to both FAMSAC and ACT Policing (910 cases in total). This was undertaken to validate the investigation outcomes recorded in the policing records and assess the court outcomes for those matters that did proceed through the criminal justice system (Table 1). There were 439 cases in which the initial investigation outcome recorded was revised during the manual case tracking review. Note that the clearance type of “not cleared” is the default clearance status applied in ACT Policing records unless it is subsequently updated.

**TABLE 1:** ACT Policing re‐categorizations.

Category	Initial clearance type	Reviewed clearance type
Proceeding		
Arrest	103	125
Charged before court	11	13
Summons	20	37
Warrant	4	0
Total	138	175
Not proceeding		
Insufficient evidence to proceed	126	237
Charge withdrawn	6	0
Cleared otherwise	15	6
Complaint withdrawn by victim	244	409
Not cleared	234	0
Offense passed to another agency	17	11
Unfounded	130	63
Pending	0	9
Total	772	735
Overall	910	910

### 2.4 Statistical analysis

Data were analyzed using STATA/BE 19.5. Descriptive statistics included frequencies for categorical variables and measures of central tendency for continuous variables. Group comparisons used linear regression to obtain *P* values via *t*‐tests for continuous outcomes and Pearson's chi‐squared tests for categorical variables. All estimates were reported with 95% confidence intervals (CIs). No potentially identifying patient characteristics are presented.

The police data refers to victims of sexual offenses with adjustments specific to this paper and therefore will not correspond with other published data such as the ACT Policing website crime statistics, ACT Policing Annual Report and other external publications.

### 2.5 ACT policing investigation outcomes definitions

Investigation outcomes were examined once the dataset of females who progressed to reporting to ACT Policing was finalized. The following definitions apply to investigation outcomes:

- *Arrest*: a suspect is arrested and taken into custody and charged.
- *Charged before court*: a suspect is charged on the back of other matters for which they are currently before the court.
- *Cleared otherwise*: an investigation has been conducted and finalized (does not fit other categories, for example the suspect was deceased).
- *Complaint withdrawn by victim*: the victim does not wish for police to proceed and withdraws the complaint.
- *Insufficient evidence to proceed*: an investigation has been conducted and is deemed as having insufficient evidence to proceed through the criminal justice system.
- *Offense passed to another agency*: a reported offense may be more appropriately dealt with by another agency. For example, ACT Policing may transfer a case for resolution to WorkSafe ACT, the independent statutory regulator responsible for enforcing compliance with ACT Work Health and Safety laws.^24^ This category also includes an offense that occurred interstate and is referred to another state/territory police force.
- *Pending*: the police investigation remains ongoing.
- *Summons*: a suspect has been summonsed for an offense(s) to appear in court at a later date.
- *Unfounded*: a report where, following an investigation of the reported circumstances, no offense is identified. For example, during a police investigation, evidence is discovered disproving the complainant's version of events leading to police having reasonable grounds to believe the reported offense did not occur.

### 2.6 ACT court outcomes definitions

- *Before court*: the matter is still before the ACT court system with no result available.
- *Convicted of other charges*: the suspect was convicted of other charges (for example burglary), but not of sexual offenses.
- *DPP withdrew charges*: DPP withdrew the charges prior to the matter being heard at court.
- *DPP withdrew charges* (*victim request*): DPP withdrew the charges at the victim's request prior to the matter being heard at court.
- *Guilty plea*: the suspect pled guilty to the charges prior to the matter being heard at trial.
- *Judge alone trial*: the matter was heard at court, whereby the Judge was the trier of fact.
- *Jury trial*: the matter was heard at court, whereby a jury was the trier of fact.

## 3 RESULTS

Figure 1 depicts the investigation outcomes as well as the court outcomes where a matter proceeded to court. Of the 2179 females who reported to FAMSAC, 910 (41.8%) also reported to ACT Policing and 175 (19.2%) proceeded to the ACT Courts.

**FIGURE 1:**
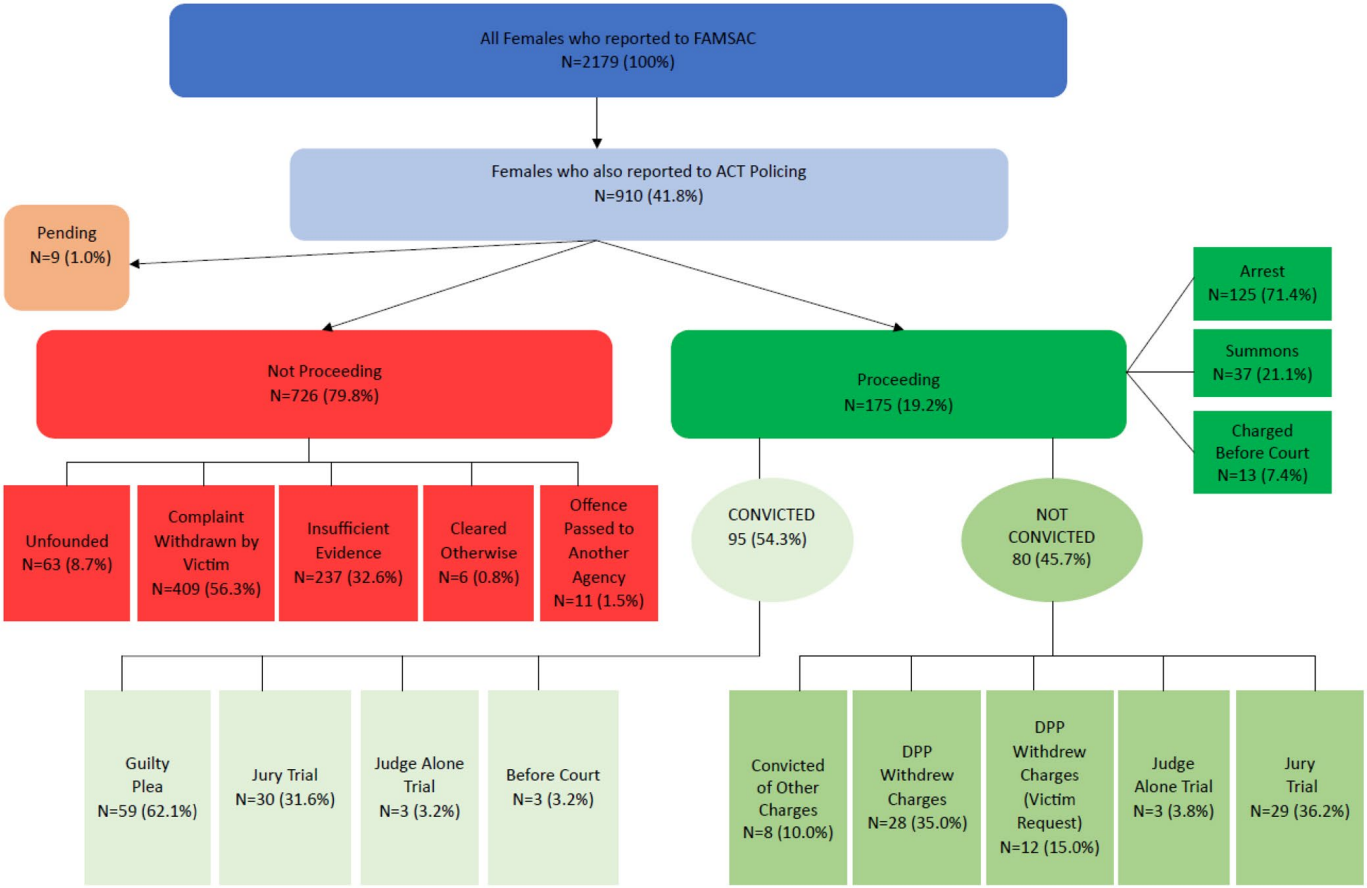
Flow chart of legal outcomes from Forensic and Medical Sexual Assault Care (FAMSAC) patients who presented to Australian Capital Territory (ACT) Policing and progression through the judicial process.

The total case number of 2179 includes females who attended for medical care only (540/2179) and historical cases where the patient presented more than 7 days after the reported assault (289/2179). It was not possible to link cases that were transferred to jurisdictions outside the ACT (514/2179), so these cases have not been included in the subsequent analysis.

In cases where the offense was passed to another agency, 11 (1.5%) did not proceed within the criminal justice system, and any resulting penalties, such as employment dismissal or sanctions imposed by immigration authorities, may have occurred but were not accessible.

The demographic characteristics of females who presented to FAMSAC, proceeded to ACT Policing, and then proceeded to the ODPP are detailed in Table 2. These have been separated into those whose matters did proceed to court (19.2%) and those whose did not (79.8%). Of 901 cases, the consumption of drugs (14.9%) had a negative effect on statistical association with case progression, whereas alcohol (57%) did not. Pre‐existing mental health conditions (54.5%) showed no statistically significant association with case progression. There were no other statistically significant associations concerning case progression with ACT Policing. The female victims whose matters proceeded to court were separated into matters where the suspect was convicted (54.3%) and matters where they were not (45.7%). There were no statistically significant associations in the judicial outcomes of conviction versus non conviction for cases proceeding to court. Genital injuries were classified as bruises, abrasions, lacerations, and redness. Non‐genital injuries were classified as bruises, abrasions, lacerations, incisions, and redness.

**TABLE 2:** Cases outcomes from ACT Policing and subsequent Office of Public Prosecutions.

	Total	Not proceeding	Proceeding to court	*P* value	Conviction	Not convicted	*P* value	Case withdrawn	*P* value
Variable	901 (100%)	726 (80.6%)	175 (19.4%)		95 (54.3%)	80 (45.7%)		409 (45.4%)	
Age group									
13–14	43 (4.8%)	32 (74.4%)	11 (25.6%)	0.608	4 (36.4%)	7 (63.6%)	0.701	18 (4.4%)	0.587
15–16	109 (12.1%)	92 (84.4%)	17 (15.6%)		8 (47.1%)	9 (52.9%)		70 (17.1%)	
17–18	135 (15.0%)	107 (79.3%)	28 (20.7%)		13 (46.4%)	15 (53.6%)		60 (14.7%)	
19–21	166 (18.4%)	135 (81.3%)	31 (18.7%)		20 (64.5%)	11 (35.5%)		78 (19.1%)	
22–25	147 (16.3%)	110 (74.8%)	37 (25.2%)		20 (54.1%)	17 (45.9%)		62 (15.2%)	
26–30	108 (12.0%)	91 (84.3%)	17 (15.7%)		8 (47.1%)	9 (52.9%)		54 (13.2%)	
31–40	107 (11.9%)	89 (83.2%)	18 (16.8%)		12 (66.7%)	6 (33.3%)		44 (10.8%)	
41–50	64 (7.1%)	51 (79.7%)	13 (20.3%)		8 (61.5%)	5 (38.5%)		19 (4.6%)	
51–59	15 (1.7%)	13 (86.7%)	2 (13.3%)		1 (50.0%)	1 (50.0%)		3 (0.7%)	
60 and over	7 (0.8%)	6 (85.7%)	1 (14.3%)		1 (100.0%)	0 (0.0%)		1 (0.2%)	
Employment status									
Employed	344 (38.2%)	284 (82.6%)	60 (17.4%)	0.653	32 (53.3%)	28 (46.7%)	0.773	141 (34.5%)	0.347
Unemployed	224 (24.9%)	178 (79.5%)	46 (20.5%)		26 (56.5%)	20 (43.5%)		115 (28.1%)	
Student	258 (28.6%)	207 (80.2%)	51 (19.8%)		26 (51.0%)	25 (49.0%)		129 (31.5%)	
Home duties	29 (3.2%)	21 (72.4%)	8 (27.6%)		6 (75.0%)	2 (25.0%)		12 (2.9%)	
*Missing*	*46* (*5.1%*)	*36* (*78.3%*)	*10* (*21.7%*)		*5* (*50.0%*)	*5* (*50.0%*)		*12* (*2.9%*)	
Victim consumed illicit substances									
Yes	134 (14.9%)	114 (85.1%)	20 (14.9%)	0.028	11 (55.0%)	9 (45.0%)	0.969	73 (17.8%)	0.045
No	690 (76.6%)	558 (80.9%)	132 (19.1%)		71 (53.8%)	61 (46.2%)		308 (75.3%)	
Unsure	77 (8.5%)	54 (70.1%)	23 (29.9%)		13 (56.5%)	10 (43.5%)		28 (6.8%)	
Victim consumed alcohol									
Yes	514 (57.0%)	412 (80.2%)	102 (19.8%)	0.925	54 (52.9%)	48 (47.1%)	0.837	210 (51.3%)	0.290
No	332 (36.8%)	269 (81.0%)	63 (19.0%)		36 (57.1%)	27 (42.9%)		178 (43.5%)	
Unsure	55 (6.1%)	45 (81.8%)	10 (18.2%)		5 (50.0%)	5 (50.0%)		21 (5.1%)	
Mental health condition									
Yes	491 (54.5%)	394 (80.2%)	97 (19.8%)	0.952	48 (49.5%)	49 (50.5%)	0.264	235 (57.5%)	0.066
No	404 (44.8%)	327 (80.9%)	77 (19.1%)		46 (59.7%)	31 (40.3%)		171 (41.8%)	
*Missing*	*6* (*0.7%*)	*5* (*83.3%*)	*1* (*16.7%*)		*1* (*100.0%*)	*0* (*0.0%*)		3 (0.7%)	
Day of assault									
Sunday	196 (21.8%)	149 (76.0%)	47 (24.0%)	0.167	24 (51.1%)	23 (48.9%)	0.659	83 (20.3%)	0.071
Monday	85 (9.4%)	62 (72.9%)	23 (27.1%)		11 (47.8%)	12 (52.2%)		39 (9.5%)	
Tuesday	47 (5.2%)	42 (89.4%)	5 (10.6%)		2 (40.0%)	3 (60.0%)		35 (8.6%)	
Wednesday	88 (9.8%)	69 (78.4%)	19 (21.6%)		11 (57.9%)	8 (42.1%)		48 (11.7%)	
Thursday	87 (9.7%)	75 (86.2%)	12 (13.8%)		4 (33.3%)	8 (66.7%)		46 (11.2%)	
Friday	143 (15.9%)	119 (83.2%)	24 (16.8%)		14 (58.3%)	10 (41.7%)		56 (13.7%)	
Saturday	193 (21.4%)	160 (82.9%)	33 (17.1%)		21 (63.6%)	12 (36.4%)		81 (19.8%)	
Multiple	22 (2.4%)	17 (77.3%)	5 (22.7%)		4 (80.0%)	1 (20.0%)		0 (0.0%)	
*Missing*	*40* (*4.4%*)	*33* (*82.5%*)	*7* (*17.5%*)		*4* (*57.1%*)	*3* (*42.9%*)		*3* (*0.7%*)	
Assault to presentation time									
< 12 h	297 (33.0%)	231 (77.8%)	66 (22.2%)	0.772	33 (50.0%)	33 (50.0%)	0.648	146 (35.7%)	0.515
12–24 h	208 (23.1%)	172 (82.7%)	36 (17.3%)		22 (61.1%)	14 (38.9%)		87 (21.3%)	
25–72 h	180 (20.0%)	148 (82.2%)	32 (17.8%)		20 (62.5%)	12 (37.5%)		81 (19.8%)	
73 h to 7 days	87 (9.7%)	70 (80.5%)	17 (19.5%)		9 (52.9%)	8 (47.1%)		39 (9.5%)	
>7 days	115 (12.8%)	94 (81.7%)	21 (18.3%)		9 (42.9%)	12 (57.1%)		51 (12.5%)	
*Missing*	*14* (*1.6%*)	*11* (*78.6%*)	*3* (*21.4%*)		*2* (*66.7%*)	*1* (*33.3%*)		*5* (*1.2%*)	
Victim genital injury									
Yes	227 (25.2%)	186 (81.9%)	41 (18.1%)	0.764	20 (48.8%)	21 (51.2%)	0.63	104 (25.4%)	0.760
No	428 (47.5%)	345 (80.6%)	83 (19.4%)		45 (54.2%)	38 (45.8%)		213 (52.1%)	
No examination	246 (27.3%)	195 (79.3%)	51 (20.7%)		30 (58.8%)	21 (41.2%)		92 (22.5%)	
Victim non‐genital injury									
Yes	369 (41.0%)	298 (80.8%)	71 (19.2%)	0.613	41 (57.7%)	30 (42.3%)	0.105	156 (38.1%)	0.150
No	299 (33.2%)	245 (81.9%)	54 (18.1%)		23 (42.6%)	31 (57.4%)		156 (38.1%)	
No examination	233 (25.9%)	183 (78.5%)	50 (21.5%)		31 (62.0%)	19 (38.0%)		97 (23.7%)	
Oral penetration									
Yes	206 (22.9%)	172 (83.5%)	34 (16.5%)	0.649	13 (38.2%)	21 (61.8%)	0.197	92 (22.5%)	0.614
No	447 (49.6%)	356 (79.6%)	91 (20.4%)		54 (59.3%)	37 (40.7%)		234 (57.2%)	
Unsure	236 (26.2%)	189 (80.1%)	47 (19.9%)		26 (55.3%)	21 (44.7%)		77 (18.8%)	
*Missing*	*12* (*1.3%*)	*9* (*75.0%*)	*3* (*25.0%*)		*2* (*66.7%*)	*1* (*33.3%*)		*6* (*1.5%*)	
Vaginal penetration									
Yes	628 (69.7%)	508 (80.9%)	120 (19.1%)	0.951	63 (52.5%)	57 (47.5%)	0.632	318 (77.8%)	0.651
No	42 (4.7%)	34 (81.0%)	8 (19.0%)		6 (75.0%)	2 (25.0%)		18 (4.4%)	
Unsure	219 (24.3%)	175 (79.9%)	44 (20.1%)		24 (54.5%)	20 (45.5%)		67 (16.4%)	
*Missing*	*12* (*1.3%*)	*9* (*75.0%*)	*3* (*25.0%*)		*2* (*66.7%*)	*1* (*33.3%*)		*6* (*1.5%*)	
Anal penetration									
Yes	137 (15.2%)	117 (85.4%)	20 (14.6%)	0.441	11 (55.0%)	9 (45.0%)	0.951	47 (11.5%)	0.027
No	523 (58.0%)	419 (80.1%)	104 (19.9%)		55 (52.9%)	49 (47.1%)		280 (68.5%)	
Unsure	229 (25.4%)	181 (79.0%)	48 (21.0%)		27 (56.3%)	21 (43.8%)		76 (18.6%)	
*Missing*	*12* (*1.3%*)	*9* (*75.0%*)	*3* (*25.0%*)		*2* (*66.7%*)	*1* (*33.3%*)		*6* (*1.5%*)	
Non‐fatal strangulation									
Yes	86 (9.5%)	72 (83.7%)	14 (16.3%)	0.799	6 (42.9%)	8 (57.1%)	0.842	52 (12.7%)	0.272
No	400 (44.4%)	320 (80.0%)	80 (20.0%)		44 (55.0%)	36 (45.0%)		197 (48.2%)	
No examination	15 (1.7%)	13 (86.7%)	2 (13.3%)		1 (50.0%)	1 (50.0%)		7 (1.7%)	
*Missing*	*400* (*44.4%*)	*321* (*80.3%*)	*79* (*19.8%*)		*44* (*55.7%*)	*35* (*44.3%*)		*153* (*37.4%*)	
No of assailant									
1	730 (81.0%)	589 (80.7%)	141 (19.3%)	0.269	77 (54.6%)	64 (45.4%)	0.429	347 (84.8%)	0.112
2	54 (6.0%)	46 (85.2%)	8 (14.8%)		3 (37.5%)	5 (62.5%)		28 (6.8%)	
3	19 (2.1%)	17 (89.5%)	2 (10.5%)		2 (100.0%)	0 (0.0%)		8 (2.0%)	
>3	11 (1.2%)	10 (90.9%)	1 (9.1%)		0 (0.0%)	1 (100.0%)		8 (2.0%)	
Unknown	87 (9.7%)	64 (73.6%)	23 (26.4%)		13 (56.5%)	10 (43.5%)		18 (4.4%)	
Assailant gender									
Male	803 (89.1%)	653 (81.3%)	150 (18.7%)	0.271	81 (54.0%)	69 (46.0%)	0.968	389 (95.1%)	0.948
Female	7 (0.8%)	5 (71.4%)	2 (28.6%)		1 (50.0%)	1 (50.0%)		1 (0.2%)	
Both M & F	3 (0.3%)	3 (100.0%)	0 (0.0%)		0 (0.0%)	0 (0.0%)		0 (0.0%)	
Unknown	88 (9.8%)	65 (73.9%)	23 (26.1%)		13 (56.5%)	10 (43.5%)		19 (4.6%)	
Assailant relationship to victim									
Acquaintance	332 (36.8%)	279 (84.0%)	53 (16.0%)	0.506	23 (43.4%)	30 (56.6%)	0.088	142 (34.7%)	0.096
Stranger	218 (24.2%)	173 (79.4%)	45 (20.6%)		25 (55.6%)	20 (44.4%)		126 (30.8%)	
Ex‐partner	55 (6.1%)	44 (80.0%)	11 (20.0%)		5 (45.5%)	6 (54.5%)		36 (8.8%)	
Partner	40 (4.4%)	32 (80.0%)	8 (20.0%)		8 (100.0%)	0 (0.0%)		20 (4.9%)	
Family member	24 (2.7%)	18 (75.0%)	6 (25.0%)		3 (50.0%)	3 (50.0%)		9 (2.2%)	
Unknown	85 (9.4%)	63 (74.1%)	22 (25.9%)		12 (54.5%)	10 (45.5%)		17 (4.2%)	
*Missing*	*147* (*16.3%*)	*117* (*79.6%*)	*30* (*20.4%*)		*19* (*63.3%*)	*11* (*36.7%*)		*59* (*14.4%*)	
Number of presentations									
1	803 (89.1%)	644 (80.2%)	159 (19.8%)	0.972	89 (56.0%)	70 (44.0%)	0.366	362 (88.5%)	0.867
2	57 (6.3%)	47 (82.5%)	10 (17.5%)		4 (40.0%)	6 (60.0%)		26 (6.4%)	
3	17 (1.9%)	15 (88.2%)	2 (11.8%)		1 (50.0%)	1 (50.0%)		14 (3.4%)	
4	10 (1.1%)	8 (80.0%)	2 (20.0%)		0 (0.0%)	2 (100.0%)		2 (0.5%)	
5	7 (0.8%)	6 (85.7%)	1 (14.3%)		1 (100.0%)	0 (0.0%)		1 (0.2%)	
6	4 (0.4%)	3 (75.0%)	1 (25.0%)		0 (0.0%)	1 (100.0%)		1 (0.2%)	
7	2 (0.2%)	2 (100.0%)	0 (0.0%)		0 (0.0%)	0 (0.0%)		3 (0.7%)	
8	1 (0.1%)	1 (100.0%)	0 (0.0%)		0 (0.0%)	0 (0.0%)		0 (0.0%)	
Examination attended									
Yes	682 (75.7%)	554 (81.2%)	128 (18.8%)	0.381	67 (52.3%)	61 (47.7%)	0.395	327 (80.0%)	0.324
No	219 (24.3%)	172 (78.5%)	47 (21.5%)		28 (59.6%)	19 (40.4%)		82 (20.0%)	
Forensic photography									
Yes	172 (19.1%)	139 (80.8%)	33 (19.2%)	0.745	21 (63.6%)	12 (36.4%)	0.441	82 (20.0%)	0.032
No	644 (71.5%)	515 (80.0%)	129 (20.0%)		68 (52.7%)	61 (47.3%)		318 (77.8%)	
Not protocol Previously	84 (9.3%)	71 (84.5%)	13 (15.5%)		6 (46.2%)	7 (53.8%)		9 (2.2%)	
*Missing*	*1* (*0.1%*)	*1* (*100.0%*)	*0* (*0.0%*)		*0* (*0.0%*)	*0* (*0.0%*)		0 (0.0%)	
Toxicology									
Yes	149 (16.5%)	125 (83.9%)	24 (16.1%)	0.502	15 (62.5%)	9 (37.5%)	0.467	66 (16.1%)	0.213
No	354 (39.3%)	281 (79.4%)	73 (20.6%)		36 (49.3%)	37 (50.7%)		191 (46.7%)	
*Missing*	*398* (*44.2%*)	*320* (*80.4%*)	*78* (*19.6%*)		*44* (*56.4%*)	*34* (*43.6%*)		152 (37.2%)	
Assessment duration									
< 1 h	125 (13.9%)	98 (78.4%)	27 (21.6%)	0.859	14 (51.9%)	13 (48.1%)	0.986	229 (56.0%)	0.025
1–2 h	493 (54.7%)	398 (80.7%)	95 (19.3%)		51 (53.7%)	44 (46.3%)		66 (16.1%)	
2–3 h	148 (16.4%)	120 (81.1%)	28 (18.9%)		16 (57.1%)	12 (42.9%)		13 (3.2%)	
3–4 h	25 (2.8%)	22 (88.0%)	3 (12.0%)		2 (66.7%)	1 (33.3%)		70 (17.1%)	
4 + h	110 (12.2%)	88 (80.0%)	22 (20.0%)		12 (54.5%)	10 (45.5%)		2 (0.5%)	
*Missing*	*0* (*0.0%*)	*0* (*0.0%*)	*0* (*0.0%*)		*0* (*0.0%*)	*0* (*0.0%*)		*29* (*7.1%*)	
Consultation outside business hours									
Yes	505 (56.0%)	403 (79.8%)	102 (20.2%)	0.507	52 (51.0%)	50 (49.0%)	0.299	238 (58.2%)	0.002
No	396 (44.0%)	323 (81.6%)	73 (18.4%)		43 (58.9%)	30 (41.1%)		171 (41.8%)	

*Note*: Using test Pearson across levels of proceeding and conviction for all variables. The color‐coding in the Table aligns with the corresponding outcomes in Figure 1.

The right‐hand columns detail the characteristics of cases that were withdrawn by the victim at the ACT Policing stage. If the victim did not consume drugs, no anal penetration, no forensic photography, assessment duration was 1–2 h, or if there was a consultation outside business hours the case was more likely to be withdrawn by the victim at the ACT Policing stage.

## 4 DISCUSSION

### 4.1 High attrition in sexual assault cases with forensic medical evidence

Many female victims chose not to report within the criminal justice system. Personal factors for not reporting include that the incident was inappropriate to report to police, that it was not a real crime, that it was not clear that harm was intended, that they had dealt with it themselves, shame and embarrassment, not wanting others to know, fear of reprisal, self‐blame, and a desire to protect others, such as children.^25^ Factors related to the legal system include that the victim did not know how to report, that they had a fear of the legal system, that the police would not believe them, think it was serious or do anything, or that they feared the legal process due to the anticipated treatment.^25^ Attrition of sexual assault matters prior to and during the criminal justice process is a significant issue, consistent with international,^26^ national^27^ and jurisdictional^28^ analysis and research. In our study, only four of 10 women who sought medical treatment for sexual assault continued on to report the offending to law enforcement. Low rates of success continue through the lifecycle of a criminal prosecution, with fewer than one in 20 women who sought medical treatment for sexual assault eventuating in a criminal conviction (4.35% or 95 convictions from 2179 cases). For those who go on to report, many then withdraw their complaints (56.3% in this study).

A sexual assault conviction requires the prosecution to prove beyond reasonable doubt that a sexual act occurred, that it was done without consent and that the accused acted intentionally with knowledge or were reckless toward the absence of consent from the victim. Without this threshold, wrongful convictions may result. Indeed, the over dependence of medical evidence has resulted in unlawful convictions.^29^ On the other hand, perceived low conviction rates in female sexual assault cases may result in significant detrimental effects for the complainant such as intensifying trauma, mental health issues such as depression and anxiety, and PTSD.^30^ Furthermore, on a community level, they may result in eroded trust, discouraging reporting and leaving perpetrators to remain free risking ongoing offending.^31^ A professional, fully independent role, separate from medical services, police, courts and other agencies, can provide continuous victim support akin to the UK's Independent Sexual Violence Advisors^32^ who offer specialist, trauma‐informed support to those reporting sexual assault or rape.

### 4.2 Legal outcomes independent of victim, assault and medical consultation characteristics

In this study 10.4% of females who underwent forensic medical evidence collection had their cases result in a conviction. There were no statistically relevant associations between FAMSAC collected victim, assailant, and medical care characteristics, and the likelihood of conviction at court. The lack of association may be significant, as it is inconsistent with general perceptions of medical evidence, for example, genital and non‐genital injury and its impacts on conviction. These findings align with a legal outcomes study^12^ that found no link between convictions and victims or offense characteristics. Although the earlier study measured assailant characteristics, included both domestic and sexual violence, and involved cases without clinical documentation, the present study differs in that all cases had supporting clinical documentation and focussed solely on sexual offense matters. The finding of very limited association between the medical documentation is in keeping with other studies.^12^, ^33^

This study was unable to demonstrate a statistically significant association between medical examination particulars such as passage of time between assault and examination, collection of forensic samples and presence of physical injury. This finding is also inconsistent with any perception that the conduct of a medical examination, timely presentation for medical assistance, documentation of physical injuries, and collection of forensic samples strengthens the likelihood of either charges being laid by investigating police or a conviction resulting from the subsequent judicial process. However, the importance of objective medical examinations in corroborating a victim's testimony or other evidence was not able to be separated in the final legal outcome. Contextual analysis of sexual assault cases examining factors such as the identity of the perpetrators (known/unknown), denial of sexual contact, and use of substances with toxicology findings are areas for future investigation.^34^ Indeed this data should be compared to the broader prosecution data (i.e., all sexual assault prosecutions, not just those with FAMSAC involvement) and the collection of medical evidence. Medical evidence plays a supportive role (Figure 2), with therapeutic care remaining the foremost duty of the healthcare worker. Medical evidence serves multiple functions: providing a factual and impartial explanatory opinion evidence to the court, corroborating witness statements, and offering education to police, prosecutors and defense. This in turn can influence decisions on whether to lay charges, apply for or oppose bail, advise a guilty plea, or proceed to a contested hearing trial.

**FIGURE 2:**
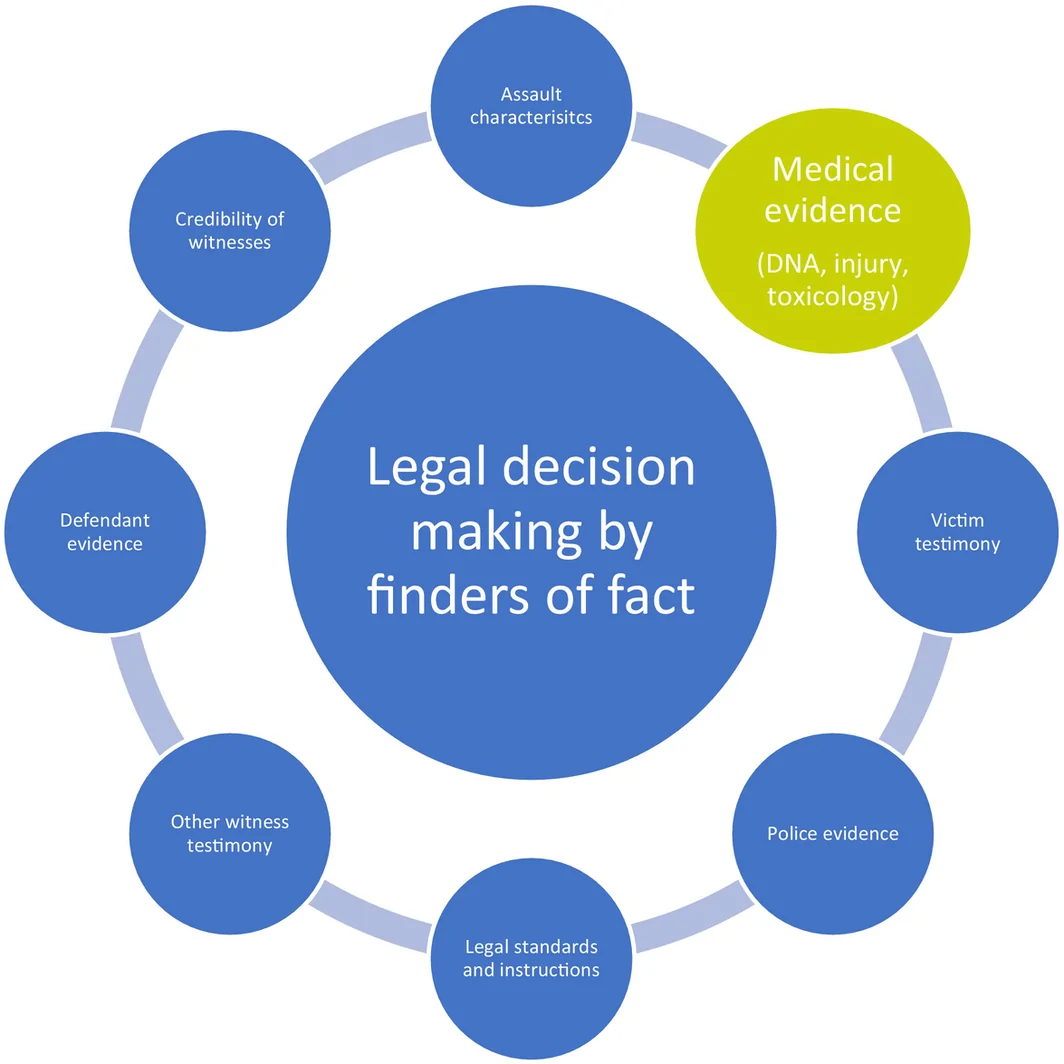
Decision making in the legal process and the role of medical evidence.

### 4.3 Factors statistically associated with police proceedings

Low overall proceedings and conviction rates are noted in this data, with more than half (56%) of the sexual assault reports made to police being withdrawn or victim contact with the investigating officers being discontinued. Of complaints reported to ACT Policing, only 19.2% resulted in criminal charges being laid. Victim withdrawal and disengagement once a matter is reported to police is the greatest driver of non‐proceedings in criminal matters.^26^, ^35^

Cases that were more likely to continue on from the police investigation stage were those involving anal penetration, the taking of forensic photography, if the victim consumed drugs, an assessment duration between 1 and 2 h, or a consultation outside business hours. Anal penetration does not appear to translate into a statistically significant prosecution outcome of conviction. Forensic photography demonstrated an association; this may also reflect the presence of injuries, as forensic photography may not be undertaken in cases with no external injury and genital photography is not undertaken by FAMSAC. Forensic photography also provides more evidence for the court to assess in its decision making. The association with consultations outside business hours may also reflect the complexity of the case and need for timely evidence collection. The authors surmise that many of the out of hours call outs that proceeded were victims who were initially referred to FAMSAC by police who had already commenced their investigation.

Cases withdrawn by the victim at the ACT Policing stage did not show a statistical association with the presence of a mental health condition (*P* value 0.066). Given that prior sexual assault increases the risk of further victimization and that sexual violence itself can lead to mental health conditions or aggravated pre‐existing mental health conditions such as depression and anxiety or PTSD,^36^ it is clear that victims with pre‐existing mental health conditions require additional support.

Perceptions that victim demographics influence the decision of police to proffer charges against an offender or for a finder of fact to make a ruling on those charges one way or the other are not supported by this statistical analysis.

### 4.4 Establishing a baseline for continuous system review

This sexual assault case tracking study provides a systematic measurement of legal outcomes from presentation to a dedicated medical and forensic sexual assault service to conviction, to establish a baseline. The applications of such a systematic measurement are to assess the effectiveness of responses to sexual violence, inform the development of a transparent accountable continual sexual assault legal service provision in the ACT, and monitor progress, thus building community trust and engagement.^30^ Tracking can be used to monitor systems improvements at each level of service provision to enhance efficiency, victim support, and the delivery of justice, for example, in combination with training programs such as Queensland's ISACURE.^37^ Importantly, consistent measurement across jurisdictions allows meaningful benchmarking, supporting comparative analyses that can highlight strengths, expose system gaps, and drive improvements in the national response to sexual violence.

A continuous and effective review of the sexual assault response system depends on strong multi‐agency coordination, because the victim journey involves multiple services, not just police. In the ACT, a single case may involve FAMSAC, ACT Policing, the ACTODPP, ACT Courts, victim‐support agencies, and broader community services such as housing and child protection. When these agencies operate in silos, information is lost and delays accumulate; the consequence is that the victim experiences fragmented care. No single agency has visibility over the entire victim journey, and each maintains its own data and definitions. Without a shared framework, it becomes impossible to track outcomes from initial presentation through to prosecution, and systemic attrition goes unaccounted for. Multi‐agency collaboration is a shared responsibility and is essential to create accountability. Low conviction rates reflect multiple decisions made by each agency, for example, FAMSAC being not able to collect specific forensic evidence resulting in ACT Policing classifying the case as unfounded and the ACTODPP unable to prosecute because of the resulting evidentiary gaps. With coordinated oversight, each agency can collectively address individual and system‐wide issues to affect attrition rates.

At the centre of this coordinated model must be the victim, who deserves a system that is accountable, integrated, and trustworthy. Such accountability is what ultimately drives cultural change, enabling visible, reviewable decisions and continuous improvement in practice.

### 4.5 Strengths and limitations

FAMSAC is the only provider of forensic medical evidence collection for adult sexual assault in the ACT and is a source of the territory's prevalence data, making it an ideal setting for case tracking population‐based outcomes for medical, police and legal interventions. Close collaborative working agreements between FAMSAC and ACT Policing have enabled this tracking study. Following stringent ethical approval, all data were de‐identified to safeguard the confidentiality of patients in the health system and females presenting to ACT Policing. Data integrity was central to this collaborative case review, to ensure robustness of the study methods, a significant proportion of outcomes from ACT Policing cases were reclassified. This case write off must be formalized for ongoing outcomes to be measured.

These data provide a baseline measurement prior to any intervention or subsequent reviews of cases and a snapshot of the past 18 years of case outcomes. Independently of this research, a review of 634 sexual assault matters between July 1, 2020 and December 31, 2021 is currently being undertaken of ACT Policing cases,^10^ with additional cases now subject to prosecution.

Generalizability to other Australian jurisdictions is limited, as the ACT is a relatively affluent, well‐educated metropolitan population that significantly differs from both smaller rural areas and metropolitan jurisdictions. Data variables such as identification as an Aboriginal or Torres Strait Islander person, ethnicity, sexuality and minority gender populations was not collected or assessed in this study, and no specific comment can be made with respect to any of these groups. Other aspects that should be addressed in future research include the level and type of intoxication with any substance, and the injury criteria which were not categorized in this study beyond the site, genital or extra genital.

### 4.6 Future research

The key unanswered question is whether presenting for forensic and medical care increases the likelihood of police action or conviction. While we tracked the female victim through the legal process, we did not examine perpetrator characteristics as other studies have done.^12^ Within the relatively contained ACT jurisdiction, this limitation can be overcome, as it is a feasible area of future research but requires data collection in a consistent way.

Conviction rates are largely driven by suspect pleas, with 62.1% of all successful ACT prosecutions arising as a result of a guilty plea by the offender and 38% of the overall conviction numbers resulting from a court trial. The benefits of early guilty pleas include a faster resolution of cases moving through the courts more quickly improving overall systems efficiency, reduction in stress and uncertainty for victims, efficient use of the court resources and sentencing discounts.^38^ However, the consequences are significant in the risk of pressure to plead,^39^ a reduced scrutiny of evidence^40^ and perception of fairness.^41^ A qualitative evaluation of early guilty pleas using this data set is both feasible and warranted. Further categorization should include determining the presence of legal aid versus private representation, exploring how guilty pleas shape case trajectories, and clarifying unintended consequences and benefits to all involved in the justice system.

This research confirms that medical findings alone do not determine case outcomes; conviction remains difficult to secure and occur regardless of medical evidence.

## 5 CONCLUSIONS

Through collaborative working practices, this study was able to establish a baseline quantitative benchmark for continuous system review in sexual assault matters from presentation within a forensic medical service to court outcome. Legal outcomes were independent of victim, assault and medical consultation characteristics. The high attrition rates occurred at all levels of service provision. This study was unable to determine any factors that associated victim characteristics, offense particulars, or aspects of the medical consultations which resulted in conviction. Medical documentation and evidence without corroborating evidence from the victim and/or other sources is not sufficient to determine the legal outcome. There are multiple factors that shape the judge and jury's decisions in sexual assault matters, and each case must be assessed on its own merits. In the context of multiple sources of evidence, medical documentation can assist the finders of fact by providing valuable insights and demonstrate alignment between the account provided and medical findings, such as injuries and toxicological results. Future research is required to determine if medical evidence increases the likelihood of police proceeding with cases, suspect pleas, and conviction.

## AUTHOR CONTRIBUTIONS

Vanita Parekh: Conceptualization, methodology, software, validation, formal analysis, investigation, resources, data curation, writing – original draft, writing – review and editing, visualization, supervision, funding acquisition. Michael Woodburn: Conceptualization, methodology, software, validation, formal analysis, investigation, resources, data curation, writing – original draft, writing – review and editing, visualization, funding acquisition. Stephaine Leonard: Writing – original draft, writing – review and editing. Bridget Bourke: Writing – original draft, writing – review and editing, visualization. Janine McMinn: Conceptualization, methodology, software, validation, formal analysis, investigation, resources, data curation, writing – original draft, writing – review and editing, visualization, funding acquisition. Anna Brkic: Conceptualization, methodology, software, validation, formal analysis, Investigation, resources, data curation, writing – original draft, writing – review and editing, visualization, project administration, funding acquisition. Ash Byron: Conceptualization, methodology, software, validation, formal analysis, investigation, resources, data curation, writing – review and editing, funding acquisition. Katelyn Pomroy: Conceptualization, methodology, software, validation, formal analysis, investigation, resources, data curation, writing – original draft, writing – review and editing, visualization, supervision, funding acquisition.

## FUNDING INFORMATION

This publication was funded by the Family Safety Community Services Directorate ACT Government, ACT Victims of Crime Commission, Canberra Hospital Private Practice Fund, ACT Policing, and an Australian Government Research Training Program Scholarship.

## CONFLICT OF INTEREST STATEMENT

All authors declare no competing interests which may have influenced the outcomes of this study.

## DEDICATION

This work is dedicated to all victim survivors of sexual assault, whose experiences may remain unheard yet deserve recognition, compassion and justice. We hope this research contributes meaningfully to their healing and to a community that values and supports them.

## Data Availability

Research data are not shared.
